# Gunshot Wounds of the Knee-Joint

**Published:** 1918-08

**Authors:** 


### *Gunshot Wounds of the Knee Joint.* By Kellogg Speed. *Journal of the American Medical Association,* March 30, 1918.

In our present series of 85 patients, the synovial lining of the joint was opened by the missile in every instance. There were two deaths. In both cases the patients suffered bone injuries at the knee, and one died from a gas infection of his arm, which was amputated, although the knee was apparently doing well. In reality, then, there was only one death caused by the knee injury, due to generalized sepsis, since no secondary operation was performed.

The synovial surface alone was involved in 42 instances, in 3 of which, or 7 o/o, amputations were made. There were bone injuries accompanying 43 knee wounds, in 6 of which, or 14 o/o, amputations were made. We might suppose, then, that a knee gunshot wound which causes simultaneous bone injury is twice as liable to go on to amputation as one without bone injury. Clinically there seems to be a higher ratio.

In the light of results of recent experience, after a knee-joint injury, the soldier should be splinted at the first dressing post and not be allowed to walk on the leg. All operations should be done at the casualty clearing stations, within 24 hours after the wound is received, when possible, or better yet, within 8 hours.

Types of knee injury to be retained at the casualty clearing station are:

(1) Those complicated by serious bone or blood vessel injury.

(2) Distended painful joint, or early signs of septic inflammation.

(3) Foreign body visible or palpable after it has opened the joint.

(4) Larger superficial wound, generally caused by shell fire, opening into the joint. Sepsis is certain to spread into the joint and immediate operation is indicated.

Early operative treatment is either radical or conservative. Radical treatment calls for either amputation or resection.

Every knee-joint gunshot wound should be operated on, if time and circumstances permit. The most innocent appearing may lead to serious trouble. The successive steps are as follows :

(1) Careful skin shaving and disinfection.

(2) The track of the missile is completely, carefully, and slowly excised with a sharp scalpel, but no scissors. No fingers or instruments are inserted through the solid wound into the joint, since not only may infection be carried in, but the foreign body may be pushed into an inaccessible area. The comminuted bone and foreign bodies are removed.

(3) The joint may be irrigated with physiologic sodium chlorid solution.

(4) The wound is closed in layers. If the synovia cannot be closed, a gauze pack is placed down to its surface.

(5) A Buck's extension is attached to a Thomas splint on the leg, and flannel bandages cover all.

Each operation takes from forty minutes to two hours.

When aseptic healing is in progress and the joint is not painful, slight passive motion may be started during the second week. Staphylococcus infection is less to be feared than streptococcus.

These wounds are excised, the synovial edge trimmed, and the joint irrigated because, without a doubt, the synovial surface is almost as well able to take care of itself as the peritoneum, if given a chance, and the resistance of the joint surface is greater than we have been led to believe. After trimming and irrigating, the operator closes the joint snugly, and its own resistance will often do the rest. Splinting prevents motion, and hence favors an early healing of the sutured synovia.

When sepsis of the joint threatens to follow conservative treatment, or no operative treatment at all, the author is inclined to side with Page, who says that no classical drainage of the knee joints is satisfactory and that the joint excision after infection is valueless. Early amputation is advisable. Long courses of joint sepsis, despite radical drainage, give too high a mortality.

SUBCRURAL POUCH DRAINAGE AND INVERSION TREATMENT : The joint is proved septic and steps should be taken at the earliest stage. Under anesthesia an opening one inch long is made in the middle of the thigh at the upper margin of the pouch. The quadriceps extension muscle fibres are separated longitudinally by a pair of May scissors, and the synovial surface of the joint is carefully opened through its upper reflection only. A medium-sized rubber tube extending just into the joint is sewed into place. The incision around the tube is left wide open to permit the secretion to run out, instead of backing into the thigh tissues. A Thomas splint and extension are applied, the left being supported by cross pieces of perforated metal both anteriorly and posteriorly, and being thoroughly padded and so bandaged that the portion over the wound can be removed for dressing without loosening the splint. The patient is then put to bed, a Balkan frame is adjusted over him, and for two hours night and morning, he is turned over on his face. Later these periods are extended until he can lie there for hours at a time.

Patients so treated should be in the early stage of joint infection, before erosion of the cartilage has taken place and before the infected contents of the joint have mechanically burst or pathologically necrosed through the synovial surface so as to set up a suppurative periarthritis.

A vertical position of the limb at right angles to the body in the Thomas splint also places the subcrural pouch in a dependent position, but the drainage is not absolute as in the prone position, and there is more tendency for progressive infection of the thigh tissues.

*Rules for Treatment of War Wounds of the Joints* : (1) At the dressing station, wounds of the joints should be immobilized with great care, in an appropriate apparatus.

(2) At the clearing station, all injured joints in which the wound is extensive, the joint tissues are lacerated or the missile is retained, and especially when a fracture is present, should be operated on, if possible, during the first six or eight hours after the patient's arrival.

(3) Roentgenoscopy is indispensable.

(4) *Operation :* Wide aseptic arthrotomy, complete exploration of the joint, systematic removal of foreign bodies. This followed by complete closure, or by closure of the capsule with superficial drainage.

(5) Resection, typical or atypical, only when there is considerable damage to the bone.

(6) *In severe suppurative arthritis* : Wide arthrotomy with complete immobilization and progressive disinfection of the wound.

In every grave case immediate resection is required.

